# Environmental Prevalence of Carbapenem Resistance Enterobacteriaceae (CRE) in a Tropical Ecosystem in India: Human Health Perspectives and Future Directives

**DOI:** 10.3390/pathogens8040174

**Published:** 2019-10-02

**Authors:** Periyasamy Sivalingam, John Poté, Kandasamy Prabakar

**Affiliations:** 1 Department F.-A. Forel for Environmental and Aquatic Sciences and Institute of Environmental Sciences, School of Earth and Environmental Sciences, Faculty of Science, University of Geneva, Uni Carl Vogt, 66 Boulevard Carl-Vogt, CH-1211 Geneva 4, Switzerland; John.pote@unige.ch; 2 Postgraduate and Research Department of Microbiology, Jamal Mohamed College, Tiruchirappalli 620020, Tamil Nadu, India; 3 Postgraduate and Research Department of Zoology, Jamal Mohamed College, Tiruchirappalli 620020, Tamil Nadu, India

**Keywords:** antibiotic resistance bacteria, CRE, environment, India

## Abstract

In the past few decades, infectious diseases have become increasingly challenging to treat, which is explained by the growing number of antibiotic-resistant bacteria. Notably, carbapenem-resistant Enterobacteriaceae (CRE) infections at global level attribute a vast, dangerous clinical threat. In most cases, there are enormous difficulties for CRE infection except a few last resort toxic drugs such as tigecycline and colistin (polymyxin E). Due to this, CRE has now been categorized as one among the three most dangerous multidrug resistance (MDR) pathogens by the US Centres for Disease Control and Prevention (CDC). Considering this, the study of the frequency of CRE infections and the characterization of CRE is an important area of research in clinical settings. However, MDR bacteria are not only present in hospitals but are spreading more and more into the environment, thereby increasing the risk of infection with resistant bacteria outside the hospital. In this context, developing countries are a global concern where environmental regulations are often insufficient. It seems likely that overcrowding, poor sanitation, socioeconomic status, and limited infrastructures contribute to the rapid spread of MDR bacteria, becoming their reservoirs in the environment. Thus, in this review, we present the occurrence of CRE and their resistance determinants in different environmental compartments in India.

## 1. Introduction 

By 2050, drug-resistant infections have been estimated to cause global economic burden and claim more mortality than cancer [1], which indicate the apparent caution. The excessive use of antibiotics in human healthcare and agriculture selects for resistance and is, therefore, the leading cause of the spreading antibiotic resistance bacteria (ARB). Of the world’s consumption, India is the leading consumer of antibiotics [2]. According to the national public health foundation, India has the highest level of infectious disease in the world and thus the consequences of antibacterial resistance could be devastating [3]. However, there is a lack of healthcare spending and is far less when compared to the per capita [3,4]. Furthermore, the World Health Organization (WHO), and India’s National Action Plan recently recognized the mitigation of antibiotic resistance in the environment as a pillar for a practical “One-health” approach to the problem [5,6]. 

The introduction of antibiotic compounds into the aquatic environment resulted in selective pressure on resident bacteria [7]. For example, some antibiotics could also be found in groundwater, deeper than 10 m [8]. It has been noted that the presence of antibiotics, even at low concentrations below the minimal inhibitory concentration (MIC), has been reported to promote various cell responses to the stress, e.g., growth arrest or cell death [9]. Besides, they can also promote horizontal gene transfer (HGT) among the microorganisms [10] and select for ARB [11]. Antibiotic resistance genes (ARGs) are frequently carried on mobile genetic elements, meaning that they can be easily transferred from one bacterium to the other. It has been reported that ARGs acquired by microorganisms could be transferred among microbes from one ecosystem to others [12]. The presence of diverse collections of resistance genes in environmental microbial communities suggests that they could be mobilized into pathogenic bacteria [13,14,15].

The effluent treatment process could be one of the major routes in the dissemination of ARB into the environment [16]. Indeed, studies have demonstrated that the microbial community in an unpolluted soil site harbours unique and ancient β-lactam resistance determinants [17,18]. Antibiotic-free (Virgin) environments are known to have several antibiotic resistance genes as housekeeping genes, and some of them share a close similarity with genes present in pathogenic microbiota [12,19,20]. Aminov (2009) also reported that the evolutionary and ecological perspective revealed that antibiotics have evolved as another way of intra- and inter-domain communication in various ecosystems [19]. Although antibiotics and resistant genes had been widely distributed in the environment before the introduction of antibiotics in clinical practice, human-driven changes have probably increased the prevalence of ARB in the water [21]. 

Ram et al. (2007) demonstrated that industrial effluents, hazardous chemicals from non-point sources of agriculture and health sectors, and inadequate cremation procedures along the banks have a serious impact on physicochemical and microbiological water quality of the river in India [22]. Consequently, the sediments, as well as the surface water, were found to have significant sources of organic carbon, phosphorus, and nitrogen. It is noteworthy that soil composition and the presence of heavy metals might enrich the antibiotic resistance genes in natural ecosystems [23,24]. Manure and sludge applied to the agricultural fields are also known to contaminate the soil and surface of food crops [25,26,27]. The soil has been reported as the largest reservoir of ARGs and is largely unstudied [28]. Soil may also act as a potential source to emerge in clinically important bacteria [29]. Hence, the understanding of the distribution and function of ARGs in soils and sediments will provide valuable information about their role in natural environments [30]. Many studies proposed that bacteria of faecal origin can be used as pollution indicators in the aquatic habitat and might be associated with the spread of infectious disease [31,32]. Besides, it may also be used to assess the clinical resistance associated with the regional level in the aquatic habitat [33,34].

It has been documented that water is one of the most important habitats on Earth and serves as a significant reservoir of antibiotic resistance [35,36]. It has also been reported that water is the primary receptor and serves as a path to disseminate bacteria from human and animal origin [37]. An impressive number of studies have demonstrated that treated and untreated urban, and hospital effluents, wastewater treatment plants (WWTP), and rainfall play an essential role in the spread of antibiotic resistance in the aquatic environment [38,39,40,41,42,43]. 

As evidenced above, the environmental compartments appear to act as a reservoir of antibiotic resistance [44]. Among the ARBs, the emergence and spread of carbapenem-resistant Enterobacteriaceae (CRE) is a potential public health risk worldwide. Though several advancements have been made to survey CRE in clinical settings, still only a very few studies have been reported from the environmental compartments worldwide. For instances, urban river ecosystem in Spain is known to have CRE with *Klebsiella pneumoniae* carbapenemase (KPC), Verona integron-encoded metallo-β-lactamase (VIM-1), and imipenemase (IMI-2) [45]. KPC-producing Enterobacteriaceae have been reported from water sampled from rivers in Portugal [46]. In Switzerland, one river was found to be positive for the presence of carbapenemase-producing (*bla*_VIM_) *Klebsiella pneumoniae* subsp. *pneumoniae* [47]. The river Danbue has also been reported to have Enterobacteriaceae harbouring New Delhi metallo-β-lactamases (NDM-1) and KPC-2 [48]. More recently, in South Korea, an urban river water sample was identified to have *Klebsiella variicola* harbouring NDM-9 [49]. 

Considering the spread of resistances in environmental compartments, it becomes important to study the distribution and frequency level of these bacteria in environments where infrastructure is often inadequate. In India, CRE was first identified in clinical settings [50], and have become increasingly reported to occur in environmental compartments which have now provoked a serious concern. This review provides insight into the regional distribution of CRE in various environmental compartments such as hospital effluents, surface water from rivers, lakes, surface sediments, and fish in India.

## 2. Carbapenem Resistance Enterobacteriaceae 

Enterobacteriaceae are belonging to the family of Gram-negative bacilli that includes the genera of *Escherichia*, *Klebsiella*, *Enterobacter*, *Serratia*, *Citrobacter*, *Proteus*, and *Morganella* [51]. Gram-negative bacteria that produce β-lactamases can inactivate β-lactams antibiotics, rendering them useless [52]. Carbapenems, a group of broad-spectrum antibiotics, are β –lactams, including imipenem, meropenem, and ertapenem, which currently are used to treat multidrug-resistant Enterobacteriaceae [53,54]. Khan et al. 2017 reported that the general mechanisms of resistance in CRE are the expression of β-lactamases, efflux pumps, loss of OprD porin, and alteration in penicillin-binding proteins (PBPs) [55].

In recent years, carbapenem-resistant Enterobacteriaceae have gained utmost importance due to having one of the least possible options to treat the infections. Mollenkopf et al. 2017 reported that CRE is emerging as a critical healthcare-associated pathogen in developed/developing nations with high fatality rates [56]. It is evident that the genes encoding resistance to carbapenem are mostly located on plasmids and associated with insertion sequences, integrons, and transposons, which facilitate their further spread [57]. The primary causative reason associated with its rapid spread is the horizontal gene transfer (HGT) mechanism among the Enterobacteriaceae [52]. There have been three main classes of carbapenemases reported, including Ambler class A ß-lactamases (KPC), class B (metallo-enzymes NDM, VIM), and class D carbapenem-hydrolysing oxacillinase (OXA-48 type) [58]. Metallo β-lactamase and Ambler class A, C, and D carbapenemases, have been differentiated based on the divalent cation and amino acid requirements for their enzymatic activity. The metallo β-lactamase does need divalent cation of Zn and serine for Ambler classes A, C, and D [59].

The global epidemiology and dissemination of CRE are mainly attributed to *KPC*, *OXA-48*, and *NDM* genes [60]. Notably, the emerging antibacterial resistance in carbapenem-resistant gram-negative bacteria, such as *bla*_NDM_, harbouring Enterobacteriaceae is one of the most challenging health concerns worldwide. Several reports emphasized that CRE harbouring an *NDM* gene isolated from hospitalized patients in the U.S., Sweden, and the UK were previously known to receive medical care in India [61,62,63,64]. Although the origin of *bla*_NDM_ is still controversial, it is spreading rapidly across countries. However, researchers raise concern that peninsular India is one among several countries endemic to NDM. To support this, some of the locally acquired NDM infections have been reported in Canada [65], China [66], France [67], Guatemala [68], Israel [69], Oman [70], Kenya [71], Kuwait [72], South Africa [73], South Korea [74], Serbia [75], and Thailand [76]. Furthermore, it should be noted that India has been reported to have the highest rate of extended-spectrum β-lactamase (ESBL) producing bacteria [77]. 

## 3. Global Clonal Type of CRE

In clinical settings, earlier reports have been documented that the occurrence of KPC-producing *K. pneumoniae* in Italy, Europe, and the USA is predominantly in sequence type ST258 [78,79,80]. On the other hand, ST11 or ST147 accounts for over 50% of NDM harbouring *K. pneumoniae* in India [81]. The endemic spread of OXA-48 associated with *K. pneumoniae* has been reported in India [82]. The sporadic spread was observed in *K. pneumoniae* harbouring OXA-48 in developed nations and mostly associated with clonal type ST101 [83,84]. Thus, the prevalence of CRE and its most dominant ST type has been well studied in clinical settings. However, there is a dearth of studies in environmental compartments. Rima Tafoukt et al. 2017 reported that different ST types among *E. coli* and *K. pneumoniae* isolated from river water in Algeria harbouring *bla*_OXA-48_. They found six different sequence types (ST) in *E. coli* including ST559, ST38, ST212, ST3541, ST1972, and ST2142 and three different ST types in *K. pneumoniae* including ST133, ST2055, and ST2192 [85]. More importantly, *K. pneumoniae* harbouring KPC-2 associated with ST437 (CC11) isolated from the river Tieteˆ and Pinheiros in Brazil [86]. In the time of globalization, the spread of ARB does not stop on one national border, as humans, animals, and food products travelling the world in a few hours are carrying their hosted CRE with them, indicating that CRE is not restricted to geographical locations [87,88,89]. 

## 4. Surface Water and Sediments as Reservoir for CRE 

### 4.1. Situation in India

**Figure 1:**
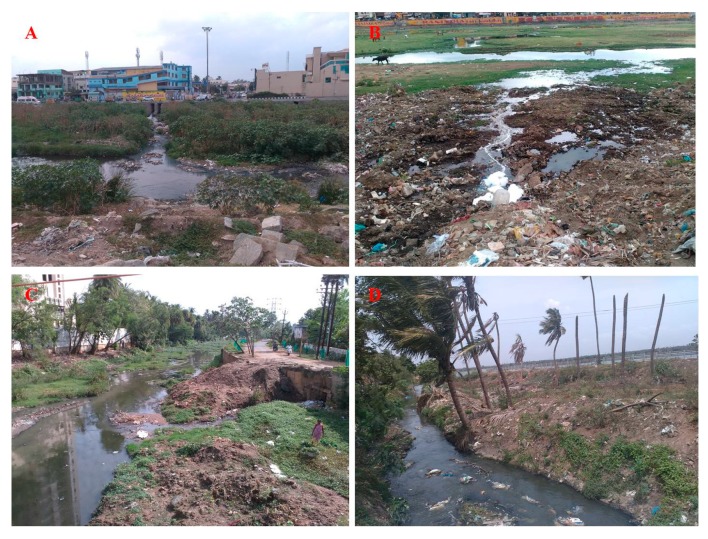
Photos show the direct discharge and impact of untreated urban effluents into the river streams and lake in the south Indian state of Tamil Nadu (**A**) river Noyyal (Tirupur); (**B**) river Vaigai (Near the Vaigai Bridge, Madurai city); (**C**) river Uyyakondan (Trichy); (**D**) lake Singanallur (Coimbatore). (**A**–**D**) photos were taken by Sivalingam in June–July 2017 (dry season).

Pathogenic bacteria which can survive and spread in the environment are of significant human health concern. Schaefer et al. 2008 reported that contamination of water bodies by anthropogenic activities cause 80% of illness and mortality in developing countries [90]. Overwhelmingly, in India alone, 60% of people do not have adequate sanitation, including toilets and no proper wastewater treatment plants (WWTP) in most of the cities [91,92]. River and lake ecosystems have been of central value to most of the Indian population by providing drinking water supply. However, more worryingly in the recent past, an increase in the discharge of untreated/partially treated urban effluents, industrial effluents, open defecation, recreational activities, and agriculture wastewater running directly into several lakes and rivers has caused a significant decrease in water quality and has tremendous impacts (Figure 1). Of note, the availability of newer generation antibiotics in pharmacies in India—over the counter sale as well as without (valid) prescription—are frequent and in some cases, carbapenem too [93,94]. The combination of the poor sanitation and the massive misuse of antibiotics in India resulted in the rapid spread of the ARB.

**Figure 2:**
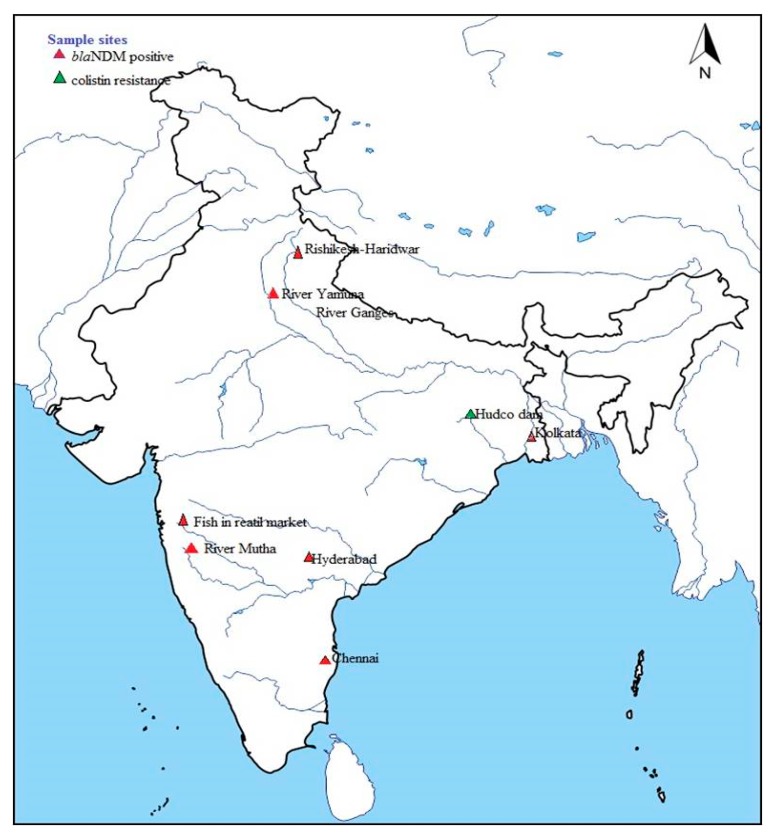
Map showing the geographical location of sampling sites and the occurrence of carbapenem-resistant Enterobacteriaceae (CRE).

ARB and ARGs found in environmental compartments are closely related to those found in clinical environments [95,96]. As a result, a recent study emphasized that the aquatic environment acts as a hot-spot for horizontal gene transfer [39]. The environmental contamination of CRE is sparsely studied both in developed and developing countries. Although the *bla*_NDM-1_ gene has been first identified in clinical settings [50], it has also been found in environmental samples such as water, soil, and sediment. However, the lack of information on these environmental compartments prevents efficient identification of human health risks. Therefore, active surveillance for CRE would be of high importance in environmental compartments in India with varying anthropogenic pressure. Until now, geographically in India, a few studies addressing the environmental prevalence of CRE have been performed at the regional level and discussed below (Figure 2).

### 4.2. Central Region

In hospital effluents from central India, the abundance of *E. coli* strains and their resistance determinants were investigated. The results showed the coexistence of ARGs, including TEM, CTXM, and OXA 48. Also, one isolate was identified to harbour NDM-1 [97]. The results are in agreement with hospital wastewater which has been proposed as one of the primary environmental sources of CRE [40].

### 4.3. Northern Region

Both drinking water and sewage samples collected from Delhi had been known to contain the *bla*_NDM-1_ gene [96]. The drinking water contamination by *bla*_NDM_ producers can be explained by the fact that water supply pipes are contaminated by public sewer sources during the rainy season. It is also evident that sediment and water samples collected in the Rishikesh-Haridwar region from the upper Ganges pilgrimage sites showed a seasonal presence of NDM genes in waters [98]. The findings suggest that the contamination might be due to a lack of adequate hygiene, unregulated disposal of wastes, discharge of partially treated wastes into upper Ganges region, and bathing during the pilgrimage season [98]. Furthermore, Bajaj et al. (2015) reported the prevalence of *E. coli* harbouring *bla*_CTX-M-15_ and *bla*_CMY-42_ in the Yamuna river (India) water samples in the city of Delhi and most of them were resistant to third-generation cephalosporins and cephamycins [77]. The discharge of urban and industrial effluents and agricultural runoff into the river could be a significant source of contamination and the selection of resistant bacteria in the water column.

Remarkably, sewage samples collected from a tertiary care hospital in northern India were found to contain *E. coli*, *Citrobacter freundii*, *Shigella boydii*, *Citrobacter braakii,* and *Citrobacter farmer*. All these species were identified to harbour *bla*_NDM_ gene. Besides, 59% of isolates were found to contain *bla*_CTX-M_ [52]. Moreover, the authors found the coexistence of several resistance determinants in the isolates such as *bla*_NDM_, *bla*_CMY_, *bla*_OXA_-type, *bla*_CTX-M_, and AmpC [52]. Although the source of *bla*_NDM_ producers is not pointed out, the authors proposed that they could have arrived from patient’s urine, sputum, and faeces into the effluents. Besides, a single plasmid type is not associated with the *bla*_NDM_ gene, as they found associations with other plasmids, such as IncFIA, IncFIB, IncP, and IncI1, most of the *E. coli* isolate was found to harbour IncFIA [52], which was corroborated with the previous report [99]. Seasonally collected effluents from the hospital, sewage treatment plants, sewer drains, and water samples from the river Yamuna in New Delhi in 2014 were found to contain CRE with *bla*_NDM-1_. The predominant species were mainly *Pseudomonas putida* followed by *Acinetobacter baumanni* and *Pseudomonas montelli* [4]. However, this is not the case in water samples collected from the Yamuna river, where the dominant species identified were in the decreasing order of *Klebsiella pneumonia*, *Klebsiella pneumoniae* subsp. *Pneumoniae*, and *Acinetobacter baumanni* [4]. Effluent samples collected from hospitals in New Delhi, 2014, during the winter period were identified to host *bla*_NDM_ harbouring Enterobacteriaceae at a higher level than when compared to that found in sewer drains. The most abundant species detected were *Klebsiella pnemoniae*, *E. coli*, *Acintobacter baumannii*, and *Pseudomonas putida* [100]. Of these studies, NDM was found to be the most common carbapenemase and indicates potential public health threat. Also, this finding suggests systematic monitoring where it has been considered as endemic. 

### 4.4. Southern Region

The high abundance of quinolone resistance bacteria in the environmental compartment in India (river) is associated with heavy discharge from antibiotic production facilities and contributes to the spread of ARGs among human pathogens [101]. Very recently, Lubbert et al. 2017 reported the extensive presence of ESBL and CRE harbouring *bla*_NDM_ and *bla*_KPC_ in the environmental samples that were contaminated by antimicrobial drug production industries [102]. It is more obvious that the antibiotic resistance genes and resistant bacteria could have been the selective effect of antibiotics. In another study, Akiba et al. 2016 reported the presence of a high abundance of CTX-M expressing *E. coli* in the water samples from the river and sewage treatment plant in India [103]. The authors also found that the occurrence of NDM harbouring *E. coli*. It was further noted that 80% of the ESBL isolates resistant to fluoroquinolones [103]. It seems likely that the identification of various carbapenemase producers may be an indication of rapid spread and evolution in the environment. 

### 4.5. Western Region

Recently, Marathe et al. 2017 reported that sediments collected from Mutha river in Pune, India, resulted positive for carbapenem resistance genes such as NDM, KPC, OXA-48, and *tet* X. Importantly, the authors have also found the presence of colistin resistance gene *mcr-1* from the upstream sediments, where there is no urban effluents discharge [54]. River sediments collected from the river Mutha in Pune, analysed by next generation sequencing NGS resulted positive for different OXA variations with reduced susceptibility to Carbapenems [15]. It is to be noted that the carbapenemase gene *bla*_NDM_ has also been identified from fish obtained in the retail market in Mumbai, India [104]. Moreover, a recent study has also indicated the occurrence of carbapenemase-harbouring *bla*_NDM_ in the seafood (fish and shellfish) in India. The identified species were *Klebsiella pneumoniae* and *Escherichia coli* [105]. The occurrence of carbapenemase-producing Enterobacteriaceae in seafood was probably due to the contamination of urban effluents into surface water and sediments, which might attribute to transfer of ARBs through food web chain. The result is highlighting the need for more studies to explore the transmission possibility of food items and public health relevance.

### 4.6. Eastern Region

Singh et al. 2017 isolated *Klebsiella pneumoniae* belonging to ST200 and ST1296 from the water samples in Hudco dam, an artificial freshwater reservoir located in Jharkhand, India. The stains were found to be positive for efflux mediated colistin resistance genes [106]. It was probably as a consequence of veterinary use of antibiotics and released into the water harvesting lake, and the selection pressure has contributed to the presence of colistin-resistant *Klebsiella pneumoniae* in the water column. In a fascinating study, Ghatak and coworkers showed that occurrence of *bla*_NDM-5_ positive *Escherichia coli* isolated from mastitic milk samples in Kolkata [107]. All these findings substantiate the community-acquired infections reported earlier by Shahid [108].

## 5. Future Directions

### 5.1. Infrastructure Development for Sanitation

Given that India is a developing country, preventive measures will significantly decrease the future spread of ARBs and ARGs in the environment. It is ascertained that the need for better management approaches and the development of appropriate urban infrastructure are on a pilot scale. To effectively control the dissemination into environmental settings, long-lasting underground sewers, waste segregation at the origin and proper disposal, coordinated public health awareness and hygiene, improved sanitation, to emphasize decreasing open defecation, are currently warranted. Towards this end, India has implemented a striking national Swachh Bharat Mission (SBM) that ensures toilets in individual homes and communities across the country. 

### 5.2. Irrigation Channels Update

Particular attention should be devoted to pilgrimage and ritual activities in the irrigation channels. In this perspective, illegal dumping of plastics, cloths, solid wastes, and open defecation should be avoided. For example, for an initiative, the river Ganges clean-up project (National Mission for Clean Ganga) is deemed to be of particular interest, and more generally, this could be extended to similar rivers in India. Despite environmental actions to rehabilitate and reduce the sources of the pollutants into the rivers and lakes, their ecological status is still poor.

### 5.3. Innovative Changes in Approach in Waste Water Treatment

Given global warming and climate change, monsoon failure over the year, and more extended periods of droughts started to occur in India. Flooding is also more frequent during a monsoon. The rising sewage inflow due to the river drying up during summer, coupled with the decreasing water availability, has affected the river’s natural cleaning process. As a result, water availability in most of the rivers and lakes is less constant. In fact, the running waters of most minor rivers and irrigational channels in urban India are primarily composed by the stagnant cesspool of filth, and untreated urban municipal, hospital, and industrial effluents and used for irrigation purposes. Therefore, innovative changes in the approach to wastewater treatment will be crucial.

### 5.4. Increased Surveillance of Food Supply Chain

Since food supply chains constitute a possible route for the spread of ARBs, national authorities should promote policies towards a more efficient way to dispose of landfills, discharge wastewater, urban solid wastes, and hospital wastes on the banks and into the river, streams, and lakes which serve in agriculture. This underscores the urgent need for systematic surveillance studies concerning ARBs and ARGs for green leafy vegetables, ready-to-eat leafy vegetables, seafood, and fish that are in direct contact with contaminated urban effluents, soil, and irrigation water at various stages, such as the harvesting and postharvest level.

### 5.5. Antimicrobials Prescription Practices/Accessibilities

There should be strict national policies implemented to curb the indiscriminate use of antibiotics in aquaculture, agriculture, veterinary, livestock farms, poultry, food animals, and antibiotic stewardship in hospitals. Moreover, it is strongly recommended that there be regulation on the sale of antibiotics at the pharmacy over-the-counter without a prescription. Although the National Action Plan to Combat Antimicrobial Resistance [5] and Chennai declaration, 2013 [109] has led to some development, there is insufficient nationwide surveillance study and effective infection control measures in the environmental compartments.

It will be of great interest to study how the climatic conditions, antibiotic residues, bacterial community, and organic load influence the persistence of CRE in aquatic ecosystems. Moreover, the increase in spending on health care, dedicated actions by different stakeholders, and involvement of non-governmental agencies (NGO) may reduce the spread of CRE and future human health risks. Besides, enhancing the engagement of neighbouring countries in active surveillance of AMR studies, will be largely helpful in tackling antibiotic resistance, and for the implementation of effective control measures.

## 6. Conclusions 

The significantly increasing number of literature presented here provides strong evidence for the distribution of CRE in the environment in India. It appears that aquatic ecosystems in India are in worrying conditions, and are identified to have CRE which might pose a severe public health concern. In this context, alarmingly, the resistance determinants identified could be potentially transferred to the microbes present in the environment. Therefore, the further widespread of CRE and evolution of resistance determinants is well foreseeable and could cause severe consequence via exposure pathways to humans. The current situation in India not only requires urgent measures to restrict the further entry of antibiotics and resistant bacteria into the water but also demands research in this area, to understand the mechanisms by which resistant bacteria can persist in the environment.
